# Supplementary material for: Development of a Novel, Potent, and Selective Sialyltransferase Inhibitor for Suppressing Cancer Metastasis
Source: Int J Mol Sci. 2024 Apr 12;25(8):4283. doi: 10.3390/ijms25084283 (PMC11050067; doi:10.3390/ijms25084283)
Supplement: Supplementary file 1 [file ijms-25-04283-s001.zip › ijms-2922673-supplementary.pdf]

Development of a Novel, Potent, and Selective Sialyltransferase Inhibitor for Suppressing Cancer Metastasis

Han-En Tsai [1], Chia-Ling Chen [1], Tzu-Ting Chang [2], Chih-Wei Fu [1,3], Wei-Chia Chen [1,4], Ser John Lynon P. Perez [1,2,5,6], Pei-Wen Hsiao [7], Ming-Hong Tai [8,9,10,*] and Wen-Shan Li [1,2,6,8,11,12,*]

1 Institute of Chemistry, Academia Sinica, Taipei 115, Taiwan; racheltsai0713@gmail.com (H.-E.T.); tt710595@gmail.com (C.-L.C.); sandisefu@gmail.com (C.-W.F.); cat85002903@gmail.com (W.-C.C.); spperez@gate.sinica.edu.tw (S.J.L.P.P.)
2 Biomedical Translation Research Center, Academia Sinica, National Biotechnology Research Park, Taipei 115, Taiwan; mrts70026@gate.sinica.edu.tw
3 Department of Chemistry, National Central University, Taoyuan 320, Taiwan
4 Department of Chemistry, National Taiwan Normal University, Taipei 106, Taiwan
5 Department of Applied Chemistry, National Yang Ming Chiao Tung University, Hsinchu 300, Taiwan
6 Sustainable Chemical Science and Technology, Taiwan International Graduate Program, National Yang Ming Chiao Tung University and Academia Sinica, Taipei 115, Taiwan
7 Agricultural Biotechnology Research Center, Academia Sinica, Taipei 115, Taiwan; pwhsiao@gate.sinica.edu.tw
8 Doctoral Degree Program in Marine Biotechnology, National Sun Yat-Sen University, Kaohsiung 804, Taiwan
9 Center for Neuroscience, National Sun Yat-Sen University, Kaohsiung 804, Taiwan
10 Institute of Biomedical Sciences, National Sun Yat-Sen University, Kaohsiung 804, Taiwan
11 Ph.D. Program in Drug Discovery and Development Industry, College of Pharmacy, Taipei Medical University, Taipei 110, Taiwan
12 Department of Medicinal and Applied Chemistry, College of Life Science, Kaohsiung Medical University, Kaohsiung 807, Taiwan
* Correspondence: minghongtai@gmail.com (M.-H.T.); wenshan@gate.sinica.edu.tw (W.-S.L.); Tel.: +886-7-5252000 (ext. 5816) (M.-H.T.); +886-2-55728662 (W.-S.L.)

Supplementary Information

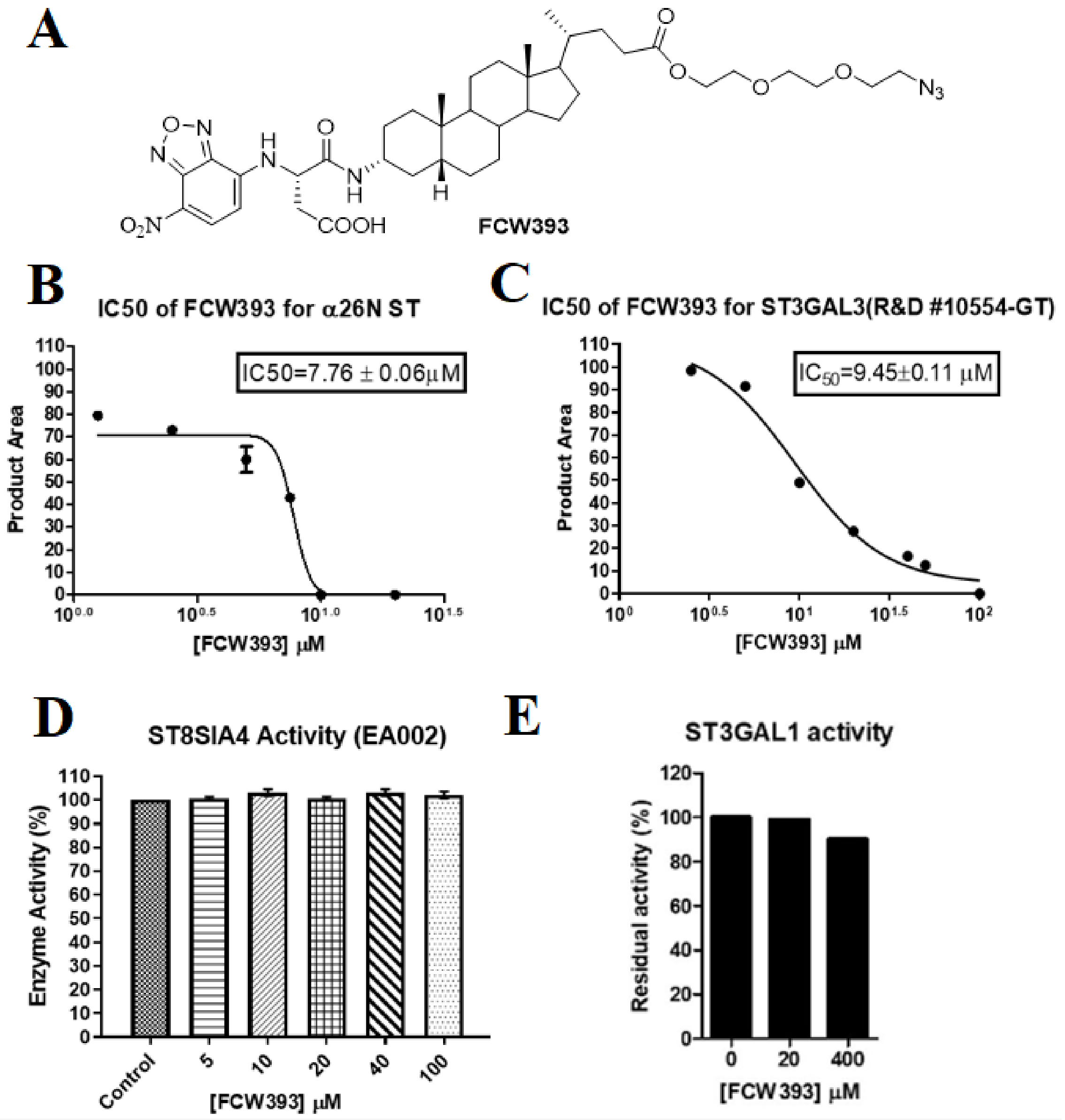

(continued)

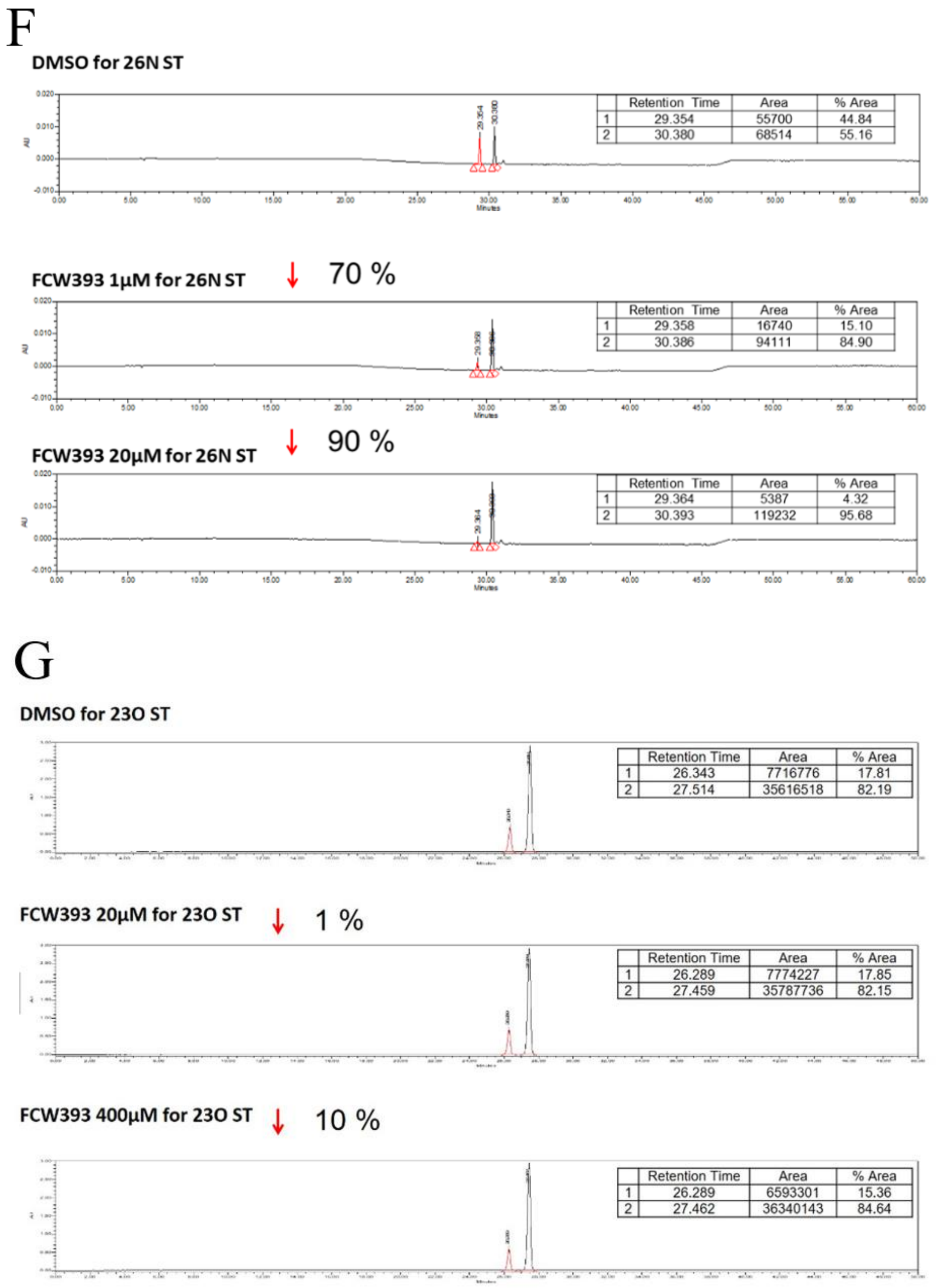

Figure S1. Sialyltransferase inhibition activity of FCW393. (A) Chemical structure of FCW393**;** (B-E) Evaluation of the sialyltransferase inhibition activity of FCW393 on (B) ST6GAL1, (C) ST3GAL3, (D) ST8Sia4 and (E) ST3GAL1 in vitro; (F-G) Representative kinetic data for the inhibition of (F)

ST6GAL1 and (G) ST3GAL1.

The *in vitro* assays for the different ST isozymes were performed as described in the methods section [1]. The data revealed that FCW393 preferentially inhibited both ST6GAL1 (α2,6-*N*-linked sialyltransferase) and ST3GAL3 (α2,3-*N*-linked sialyltransferase) activities rather than ST3GAL1 (α2,3-*O*-linked sialyltransferase) and ST8SIA4 (α2,8-*N*-linked sialyltransferase). Chemical structure of FCW393 is depicted in (A).

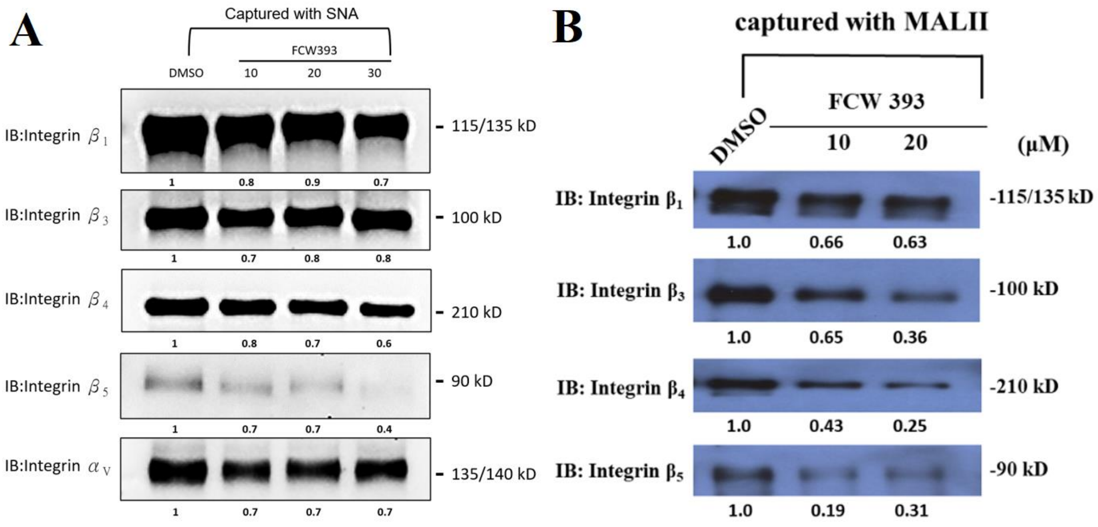

Figure S2. Inhibition of integrin sialylation by FCW393 in MDA-MB-231 cells

MDA-MB-231 cells treated with DMSO, FCW393 were harvested at 48 h after treatment. Cellular proteins were incubated with biotinylated (A) *S. nigra* lectin (SNA) or (B) *M. amurensis* lectin II (MAL II). Streptavidin-agarose beads were added to pull down sialylated proteins and the sialylation status was determined.

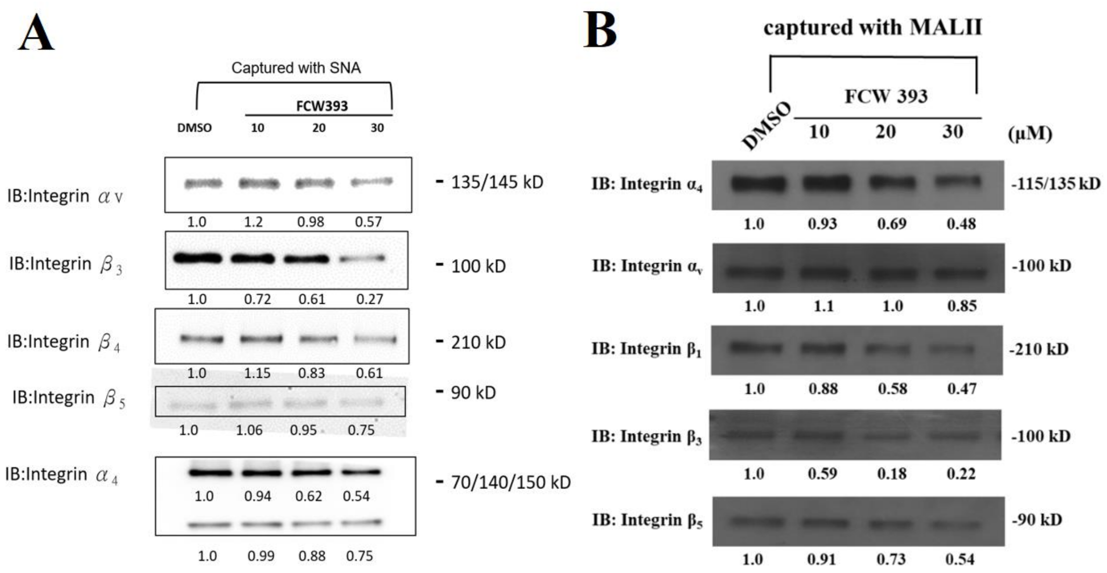

Figure S3. Inhibition of integrin sialylation by FCW393 in B16F10 cells

B16F10 cells treated with DMSO or FCW393 (0–30 μM) were harvested at 48 h after treatment. Cellular proteins were incubated with biotinylated (A) *S. nigra* lectin (SNA) or (B) *M. amurensis* lectin II (MAL II). Streptavidin-agarose beads were added to pull down sialylated proteins and the sialylation status was determined.

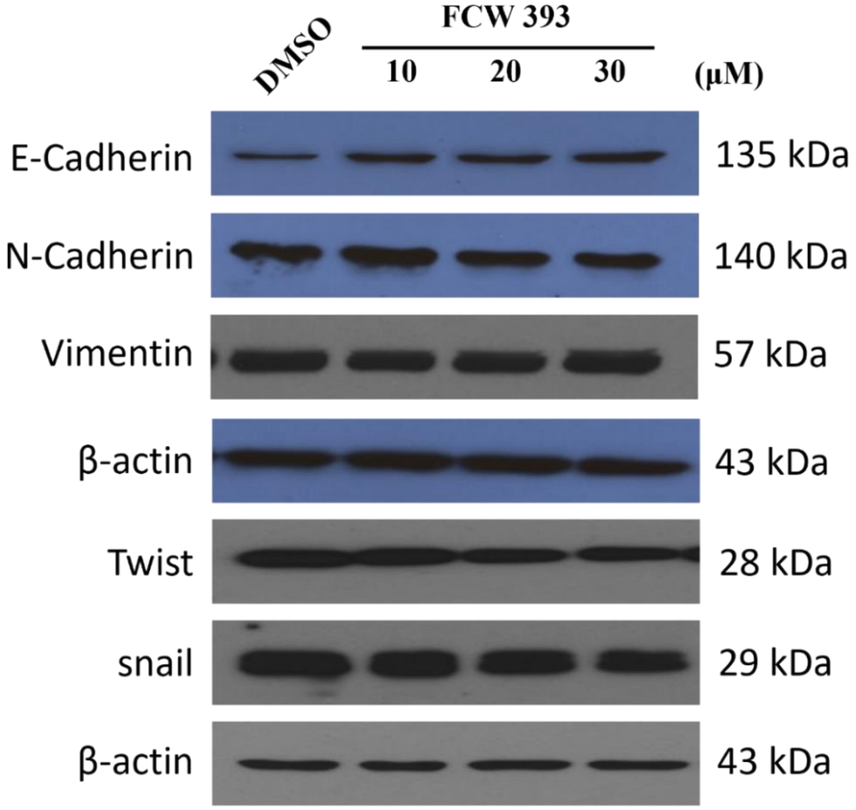

Figure S4. Effect of FCW393 treatment on the expression of EMT signaling in B16F10 melanoma cells

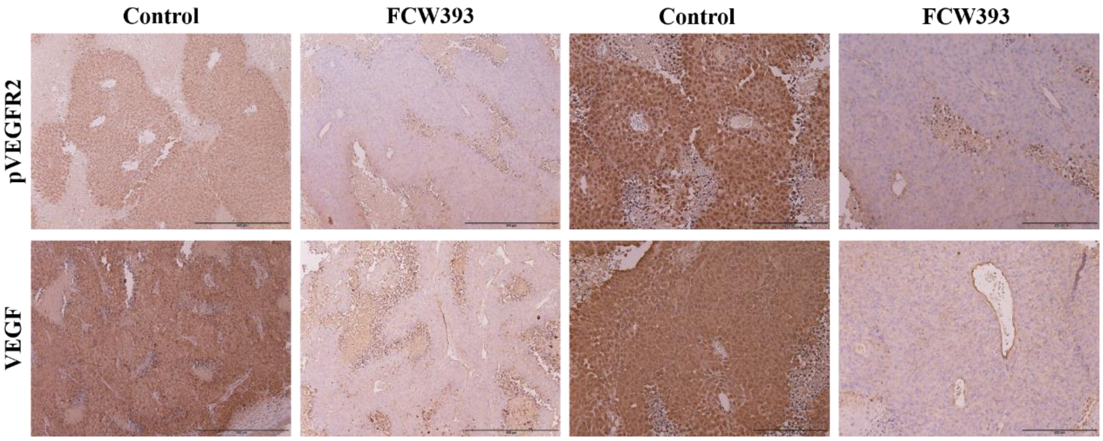

Figure S5. Effect of FCW393 on VEGFR2 and VEGF expression in B16F10 melanoma

After the mice were sacrificed, the melanoma tissues were harvested and examined by immunostaining. Immunohistochemical analysis was used to investigate *p*VEGFR2 (top) and VEGF (bottom) expression level *in vivo*.

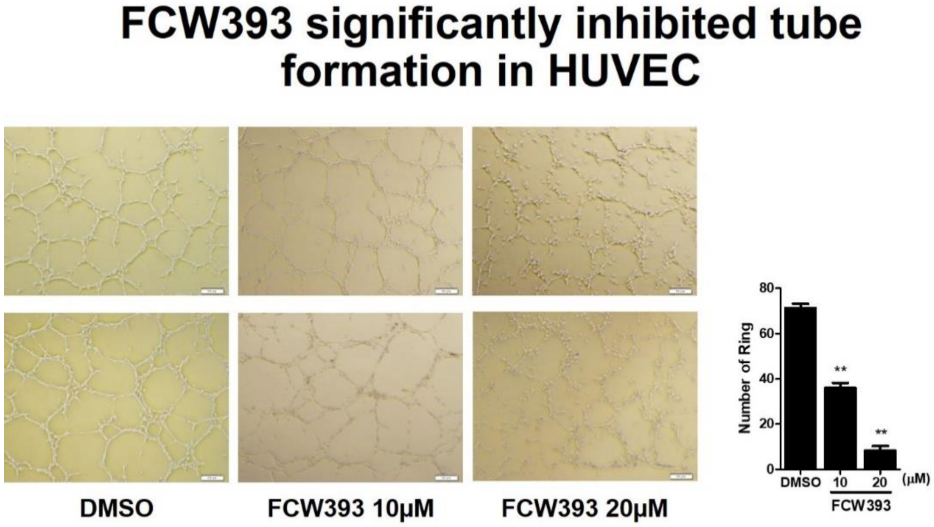

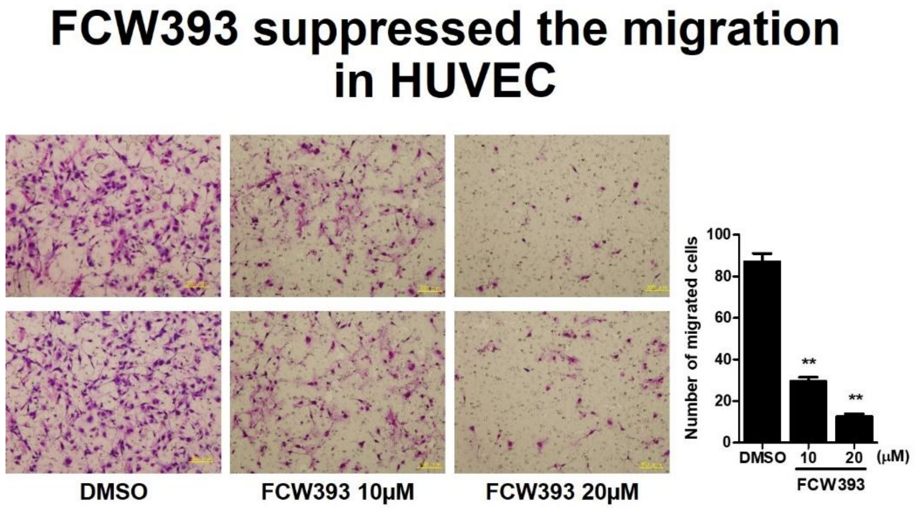

Figure S6. Effect of FCW393 treatment on the migration and tube formation of HUVECs in vitro

The *in vitro* tube formation assay was performed as described previously [2].

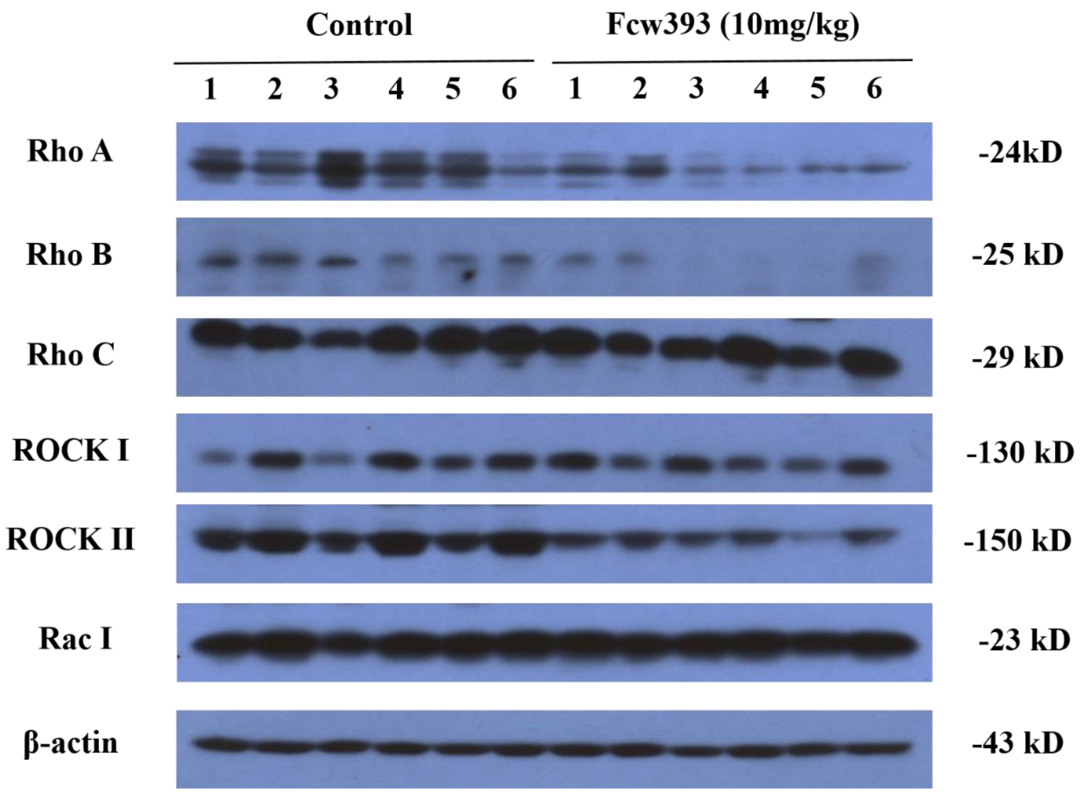

Figure S7. Effect of FCW393 on Rho-ROCK signaling in B16F10 melanoma

After the mice were sacrificed, the tumor proteins were harvested and examined by western blot. The RhoA and RhoB expression levels were inhibited by FCW393 treatment. And the Rho down-stream activation factor ROCK-II were also suppressed by FCW393 treatment (n = 6 in each group).

References:

1. Fu, C.W.; Tsai, H.E.; Chen, W.S.; Chang, T.T.; Chen, C.L.; Hsiao, P.W.; Li, W.S. Sialyltransferase Inhibitors Suppress Breast Cancer Metastasis. *J. Med. Chem.* **2021**, *64*, 527-542.
2. Chen, J.Y.; Tang, Y.A.; Huang, S.M.; Juan, H.F.; Wu, L.W.; Sun, Y.C.; Wang, S.C.; Wu, K.W.; Balraj, G.; Chang, T.T.; Li, W.S.; Cheng, H.C.; Wang, Y.C. A novel sialyltransferase inhibitor suppresses FAK/paxillin signaling and cancer angiogenesis and metastasis pathways. *Cancer Res.* **2011**, *71*, 473–483.

Scheme S1. Synthesis of compound FCW393

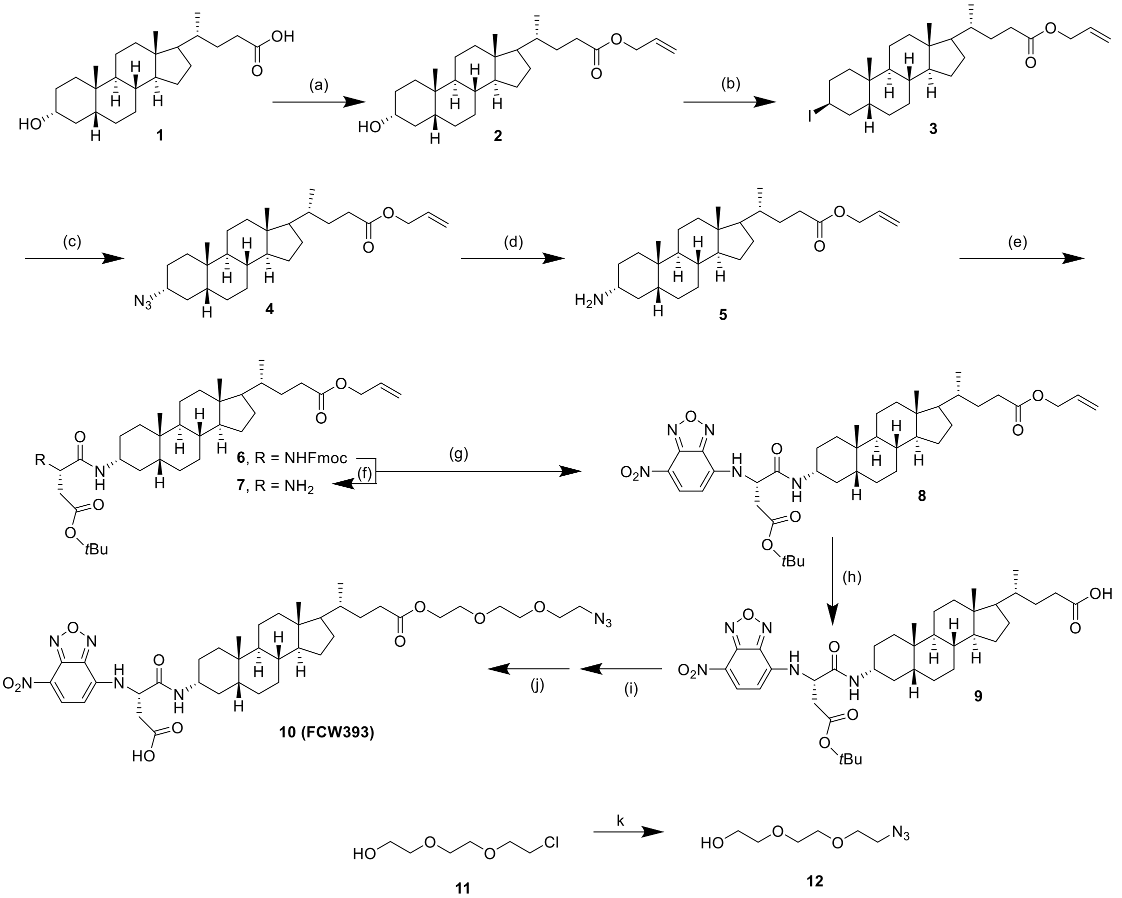

[a] Reagents and conditions: (a) allyl bromide, K_2CO_3, DMF, 100 °C, 12 h; (b) I_2, PPh_3, imidazole, CH_2Cl_2, 0 °C to rt, 12 h, 74 % (two steps); (c) NaN_3, DMF, 100 °C, 3 h, 99 %, (d) PPh_3, THF-H_2O, rt, 12 h, 72 %, (e) Fmoc-L-Asp(O-*t*Bu), HBTU, DIPEA, CH_2Cl_2, rt, 3 h, 93 %, (f) DBU, CH_2Cl_2, rt, 0.5 h, 92 %, (g) NBD-Cl, $NaHCO_3$, THF, EtOH, rt, 2 d, 42 %, (h) $Pd(PPh_3)_4$, $PhSiH_3$, CH_2Cl_2, rt, 2 h, 95 %, (i) **12**, DCC, DMAP, CH_2Cl_2, rt, 6 h, 72 %, (j) TFA, H_2O, CH_2Cl_2, rt, 3 h, 88 %.

Synthesis of **12**:

2-(2-(2-Azidoethoxy)ethoxy)ethanol (**12**)

Yield 90 % (colorless oil), ^{1}H NMR (400 MHz, $CDCl_3$): δ 3.71-3.66 (m, 4H), 3.63-3.61 (m, 4H), 3.59-3.54 (m, 3H), 3.34 (t, *J* = 4.8 Hz, 1H), 2.66-2.64 (m, 1H). ^{13}C NMR (100 MHz, $CDCl_3$): δ 72.5, 71.2, 70.5, 70.2, 61.4, 50.6. HRMS calcd for $C_6H_{14}O_3N_3$ $(M + H)^+$, 176.1035; found, 176.1034.

^{1}H- NMR of compound **12**

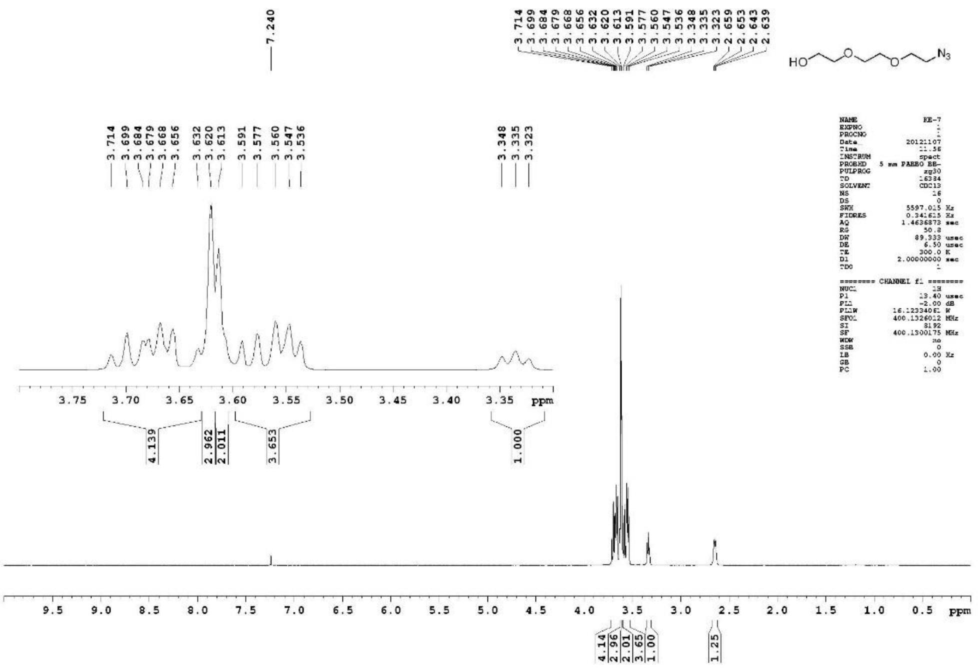

^{13}C-NMR of compound **12**

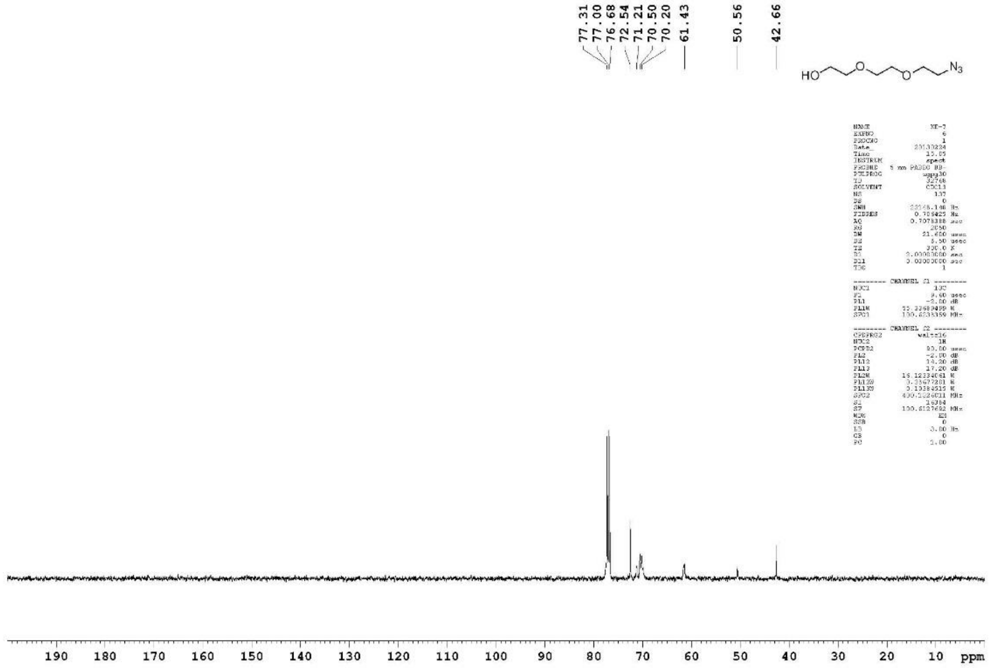

HRMS of compound **12**

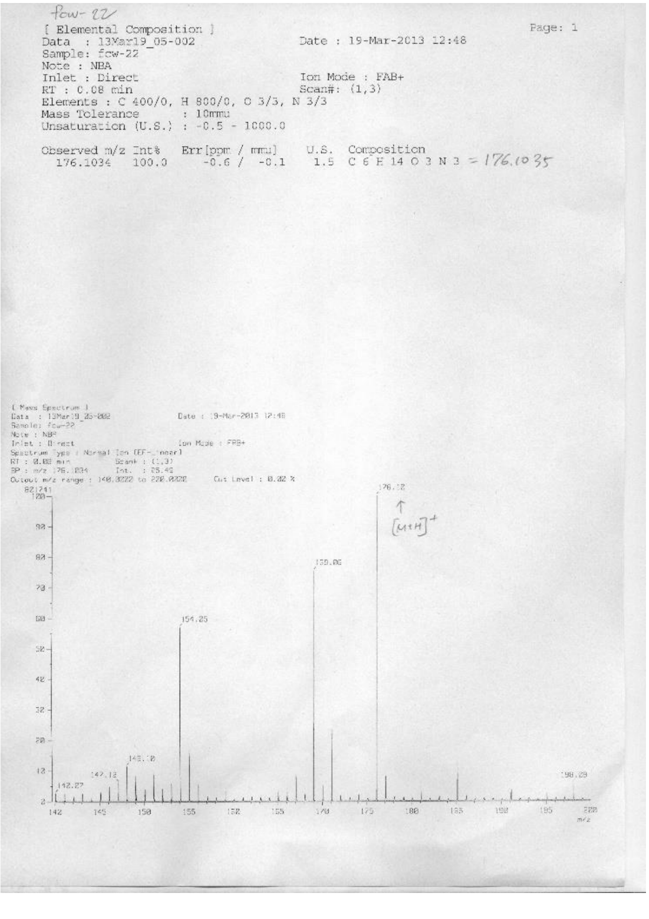

Synthesis of **FCW393**

(3S)-4-(((3R,5R,10S,13R)-17-((R)-5-(2-(2-(2-Azidoethoxy)ethoxy)ethoxy)-5-oxopentan-2-yl)-10,13-dimethylhexadecahydro-1H-cyclopenta[a]phenanthren-3-yl)amino)-3-((7-nitrobenzo[c][1,2,5]oxadiazol-4-yl)amino)-4-oxobutanoic acid (**FCW393**)

mp 80-81 °C, ^{1}H NMR (400 MHz, $CDCl_3$): δ 8.37 (s, 1H), 7.77 (s, 1H), 7.01 (s, 1H), 6.35 (s, 1H), 4.87-4.76 (m, 1H) 4.21 (s, 2H), 3.80-3.66 (m, 9H), 3.38 (s, 2H), 3.14-3.06 (m, 2H), 2.35-2.30 (m, 1H), 2.23-2.16 (m, 1H), 1.79-1.72 (m, 7H), 1.46-1.09 (m, 13H), 1.02-0.98 (m, 3H), 0.86 (s, 3H), 0.79 (d, *J* = 4.0 Hz, 3H), 0.74-0.67 (m, 3H) 0.53 (s, 3H). ^{13}C NMR (100 MHz, $CDCl_3$): δ 174.8, 173.2, 168.2, 144.3, 143.7, 142.8, 136.4, 124.5, 100.8, 70.6, 70.5, 70.0, 69.2, 63.5, 56.5, 56.1, 54.1, 50.7, 50.4, 42.6, 42.3, 40.4, 40.1, 35.7, 35.6, 35.3, 34.4, 32.9, 31.2, 30.9, 28.1, 27.3, 26.9, 26.2, 24.0, 23.4, 20.7, 18.2, 11.9. HRMS calcd for $C_{40}H_{58}N_8O_{10}Na$ $(M + Na)^+$, 833.4174; found, 833.4181. HPLC purity: ＞99.5 %.

^{1}H- NMR of compound **FCW393**

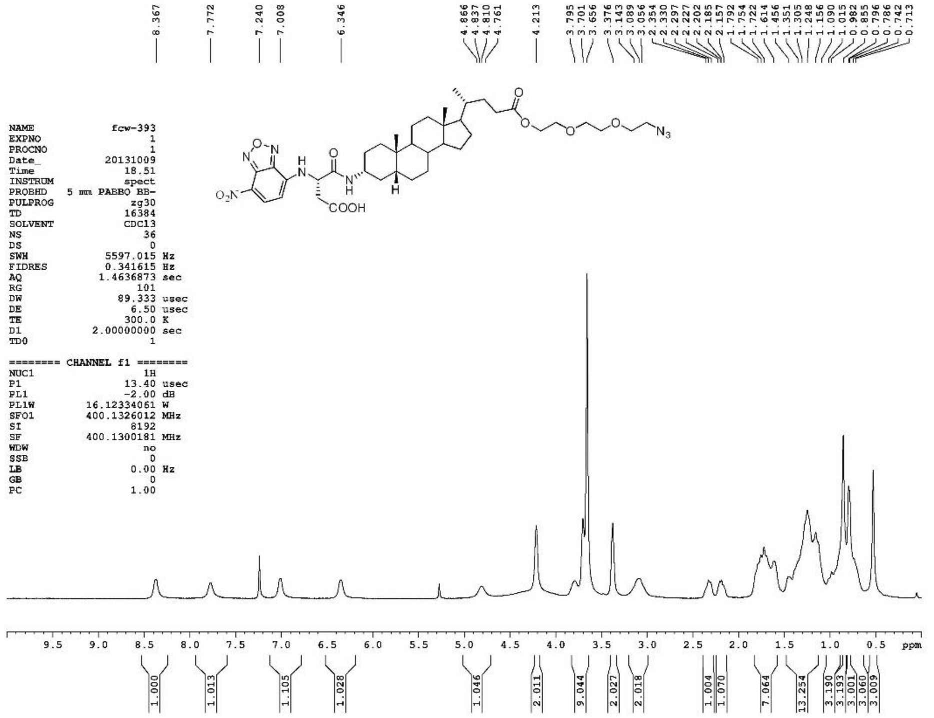

^{13}C-NMR of compound **FCW393**

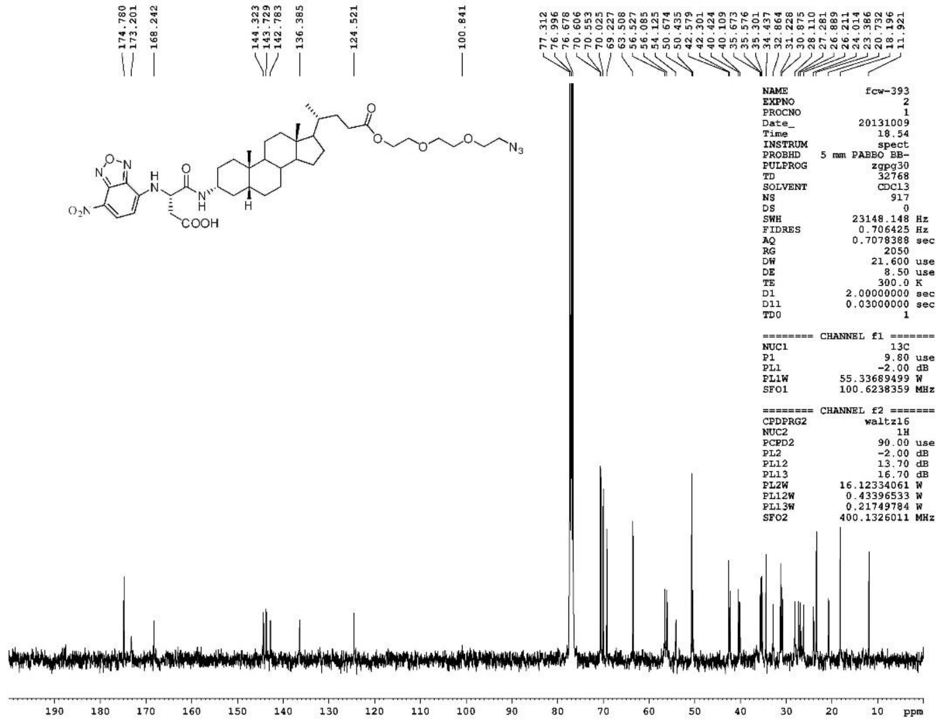

HRMS of compound **FCW393**

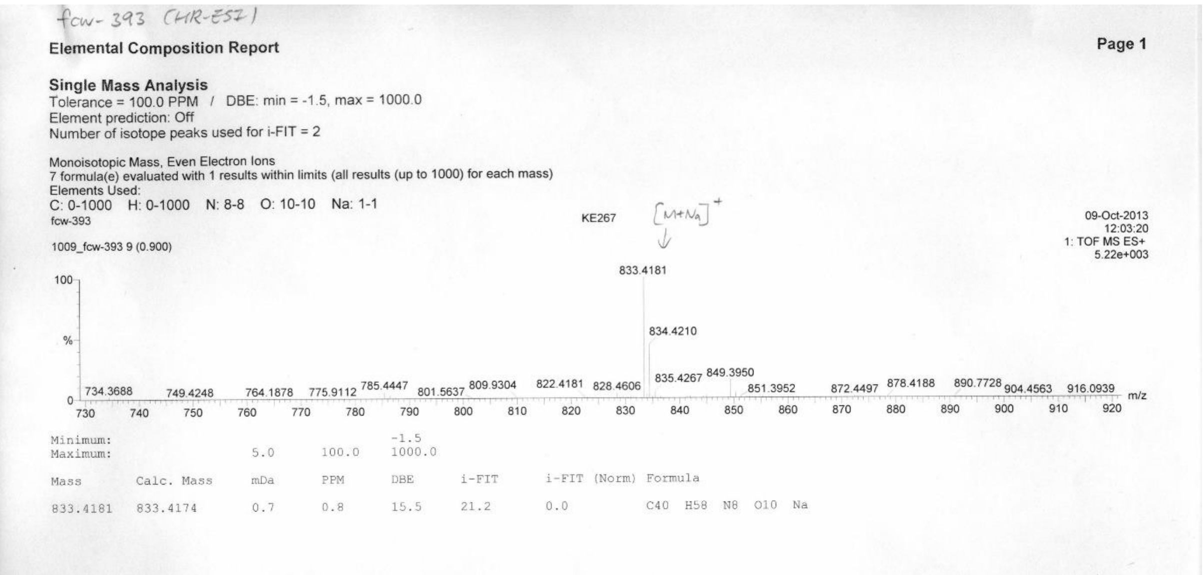
fcw-393 (HR-ESI)

Elemental Composition Report Page 1

Single Mass Analysis
Tolerance = 100.0 PPM / DBE: min = -1.5, max = 1000.0
Element prediction: Off
Number of isotope peaks used for i-FIT = 2

Monoisotopic Mass, Even Electron Ions
7 formula(e) evaluated with 1 results within limits (all results (up to 1000) for each mass)
Elements Used:
C: 0-1000 H: 0-1000 N: 8-8 O: 10-10 Na: 1-1

Mass	Calc. Mass	mDa	PPM	DBE	i-FIT	i-FIT (Norm)	Formula
Minimum:				-1.5			
Maximum:		5.0	100.0	1000.0			
833.4181	833.4174	0.7	0.8	15.5	21.2	0.0	C40 H58 N8 O10 Na

HPLC purification of compound **FCW393**

254 nm

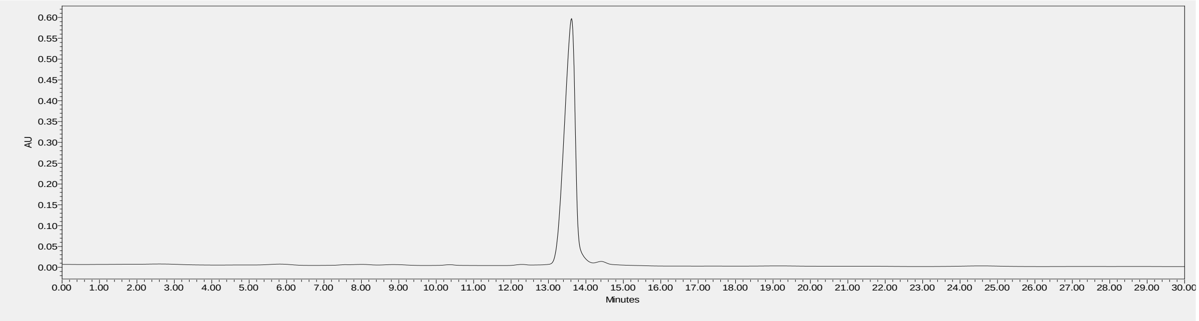

Conditions:

A: H_2O + 0.1 % TFA

B: ACN + 0.05 % TFA

Flow A: 0.45 ml/min, Flow B: 1.55 ml/min

Isocratic

Retention time: 13.62 min

Column: Kromasil 300-5C4 column
